# Work Intensification and Psychological Detachment: The Mediating Role of Job Resources in Health Service Workers

**DOI:** 10.3390/ijerph182212228

**Published:** 2021-11-21

**Authors:** Juan Sandoval-Reyes, Juan C. Restrepo-Castro, Jair Duque-Oliva

**Affiliations:** 1 Departamento de Psicología Social y las Organizaciones, Facultad de Psicología, Universidad de La Sabana, Chía 250001, Colombia; 2 Departamento de Evaluación e Intervención Psicológica, Facultad de Psicología, Universidad de La Sabana, Chía 250001, Colombia; juanreca@unisabana.edu.co; 3 Escuela de Administración y Contaduría Pública, Facultad de Ciencias Económicas, Universidad Nacional de Colombia, Bogotá 111321, Colombia; ejduqueo@unal.edu.co; 4 ESAI Business School, Universidad Espíritu Santo, Samborondon 104135, Ecuador

**Keywords:** work-related stress, psychological detachment, job resources, stress recovery, PLS-SEM

## Abstract

Psychological detachment is the central experience of recovery from work-related stress that allows individuals to reduce burnout symptoms. The stressor-detachment model (SDM) contends that job resources moderate the relationship between job stressors and psychological detachment. We designed an instrument to measure job resources from a multidimensional perspective. A sample of *n* = 394 individuals from the health service industry participated in the study. Data indicate that job resources comprise a four-factor structure underlying a formative model. Consistent with the SDM, a partial least squares structural equation modeling (PLS-SEM) analysis suggests a moderating effect of job resources (e.g., control over working conditions, leaders’ emotional support), between work intensification and psychological detachment. In addition; results indicate that workers who perceive high levels of support from their organization achieved higher levels of detachment compared with those who perceived low levels of support. Theoretical as well as practical implications for stress management practices, occupational health, and well-being are discussed.

## 1. Introduction

The workplace environment has changed rapidly over the past decades, and managerial decisions oriented towards maximizing profits are affecting working conditions as they become stressful for individuals [1]. According to Bakker [2], nowadays people must work harder while facing work overloads, and higher cognitive and emotional demands. Furthermore, work is intensified as people access information on their mobile devices, and have the possibility to work any time, any place [3].

This modern world reality poses a health problem to individuals’ well-being as work becomes a source of stress in their lives. According to Pfeffer [4], there is evidence to suggest an increase of cardiovascular diseases, substance abuse, eating and sleeping disorders as a result of work-related stress. In order to face these increasingly demanding work dynamics, people need to maintain optimal physical and psychological states, motivation, and commitment [2,5]. A mechanism to achieve the latter is stress recovery, a process by which symptoms of physical and psychological burnout caused by job demands are reduced or eliminated [6,7]. Previous studies [8] have found a positive relationship between high recovery states, with well-being, health, motivation and performance levels. 

Sonnentag [9] stated that the central experience of recovery is psychological detachment from work (PDW), which implies not thinking about work-related issues once a workday is over. According to the stressor-detachment model (SDM) [7], detachment may be understood as a mediator or moderator of the relation between a job stressor and a person’s well-being. In support for this hypothesis, the Wendsche and Lohmann-Haislah’s [10] meta-analysis reported mild correlations among these variables.

In addition, the SDM posits that job and personal resources may moderate the relationship between stressors and detachment. In this regard, workers who perceive high levels of support may be more likely to detach themselves from work. Conversely, they may feel overwhelmed by stressors when they experience lack of resources [7].

Although findings suggest that job resources correlate positively with detachment [8,10], to the best of our knowledge, the empirical evidence on the moderating effect of job resources between job stressors and psychological detachment is limited. This is because the moderating effect of job resources has been addressed, mainly from a unidimensional perspective [11,12,13]. Additionally, research on detachment has focused on individuals, not on organizations. Such focus disregards the importance of the role of organizational contexts in individuals’ recovery process [14].

Consequently, the purpose of this study was to empirically evaluate the effect of job resources on individuals’ ability to achieve PDW from a multidimensional perspective. In addition, we intended to focus on organizational, instead of individual resources. In order to attain such purpose, we developed a scale to assess the perception of the availability of job resources for detachment. So far, there are data to support a contribution of stress to PDW. Nevertheless, to the best of our knowledge, the relationship between work intensification and PDW has not yet been studied. Therefore, we conducted a partial least squares structural equation modeling (PLS-SEM) analysis to test whether job resources moderate the relationship between work intensification and PDW.

### 1.1. Job Resources and Psychological Detachment

As mentioned, the organizational context may influence the recovery process and, therefore, reaching suitable levels of detachment does not depend exclusively on individuals [7,15,16]. Some researchers have addressed context elements that influence PDW such as resorting to new ways to work (e.g., teleworking and work connectivity), perceived segmentation norm (e.g., not answering work related e-mails during off-work hours, and role boundaries) [15,16], working overtime [17,18], social support from the team [19,20], and supervisor support [21]. Wendsche and Lohmann-Haislah, [10] examined work characteristics as an antecedent of detachment (*k* = 61 studies; *n* = 28,588) and reported a positive and significant association with job resources (*r =* 0.17 social support and *r =* 0.05 job control). It is worth mentioning that the analysis addressed the resources as one-dimensional variables, which may interfere with the possibility of detecting larger effects.

According to the Job Demands Resources (JD-R) model [22], each job resource protects from stressors in specific domains (e.g., autonomy, supervisor support and team climate) and operate at different levels: the organization of work, interpersonal and social relations, and the organizational level. Moreover, job resources combined strengthen their protective effect. Underpinned by the Conservation of Resources Theory [23], the JD-R model contends that those who have at their disposal a large amount of resources, enter a “gain spiral” which facilitates access to additional resources. By contrast, those who lack access to resources are more prone to a “loss spiral” [22].

In the same vein, the Meijman and Mulder’s [24] Effort-Recovery model establishes that organizational contexts that simultaneously offer different resources, encourage people’s motivation to face stressors and reduce the generation of negative behaviors towards their work. For the abovementioned reasons, we intend to approach job resources for detachment from a multidimensional perspective. Furthermore, our argument is based on the possibility that accomplishing detachment from work involves a set of variables that interact with each other and strengthen their protective role, thereby reducing physical and psychological costs of context demands.

Based on the literature review that links job resources with PDW, we propose four resources in our model:

1. Control over working conditions. According to research, having a high degree of control generates in the person a feeling of direct influence over their work surroundings [25]. Similarly, greater levels of control reduce exposure to job demands outside the work environment, strengthens resources for recovery, and reduces the impact of stressors associated with working conditions during off-work hours [26]. As a resource, control over working conditions evaluates the level of freedom collaborators think they have in order to define the number of hours they work, the time of the day, the use of technology outside the workplace, and the level of involvement in work-related matters beyond working hours.
2. Low availability culture. As leaders are oriented towards attaining results and goals, organizations may tacitly promote the culture of “always on work mode” [7,15]. Therefore, leaders who refrain from imposing work overloads and short-notice deadlines on their collaborators, become themselves resources that facilitate psychological detachment. As a resource, low availability culture is operationalized as collaborators’ low perception about leaders’ expectations regarding their availability beyond working hours.
3. Leaders’ emotional support. Reduction of work-related stress levels is most easily achieved when leaders are focused on helping collaborators cope with competitive demands that arise at work [27]. When leaders create supportive environments, they become an important resource to ease the negative effect of job demands, which in turn favor detachment and recovery [1]. Evidence suggests that support behaviors provided by supervisors’ aid employees increase their well-being, health and productivity [28]. As a resource, leaders’ emotional support measures the perception collaborators have on leaders’ proneness to procure their well-being, as well as their openness to discuss possible conflicts in their work-life balance.
4. Leader’s role model. At some organizations, the norm may be to address work-related matters after the workday is finished; other organizations may regard such practice as unacceptable. For this reason, the way leaders segment their role boundaries may model the behavior to follow [29]. In addition, according to research, PDW is moderated by the segmentation levels defined by the group members [15]. As a resource, the leader’s role model evaluates collaborators’ perception about the way leaders become an example that rules the adequate psychological distancing after the workday.

Based on the previously stated resources we propose:

**Hypothesis** **1:** *Job resources for detachment may be ascertained multidimensionally through the reciprocal interaction amongst: (1) control over working conditions, (2) low availability culture, (3) leaders’ emotional support, and (4) leader’s role model.*

### 1.2. Work Intensification and Psychological Detachment

Understanding what interferes with detachment, and what facilitates it, has drawn researchers’ interest, particularly regarding time pressure, work overloads, and unfinished tasks [14].

The SDM postulates that job stressors increase negative activation, which in turn prevents detachment. Such negative activation, pervades physically, cognitively, and emotionally, thus maintaining systems active during off-work periods of time [7,30,31,32].

In addition to the latter, our interest is to address the phenomenon of job demands intensification as a form of work-related stress that hinders psychological detachment. As economies become increasingly globalized, organizational structures more flexible, and digital business models accelerated, the world of work has transformed, as well as organizational management practices [33]. Intensification refers to an increase in work in which progressively less time is available to devote to the same tasks. This tends to become an established, instead of an occasional demand. From this perspective, work intensification is a hindrance stressor as it demands a continuous and extra mobilization of mental and emotional resources. Such changes in work conditions are new job demands that entail multitask labor, high levels of connectivity, and the trend of being “always available”. All the previous changes call for additional empirical research [34].

From a business perspective work intensification have short-term benefits (i.e., productivity, competitiveness, and profitability). Conversely, work intensification has negative effects on collaborators. Franke [35], contends that such intensification brought about by social acceleration may be conceived as a new demand that requires individuals to increasingly use mental and emotional resources. Additionally, there is evidence that the perception of intensification may cause stress responses that are negatively related to health indicators [36,37,38]. To the best of our knowledge, there are no studies aimed to address the relationship between work intensification and psychological detachment. Therefore, we propose:

**Hypothesis** **2:** *Work intensification has a negative effect on PDW.*

### 1.3. Moderating Effects of Job Resources

As previously mentioned, the SDM asserts that the effect of job stressors on detachment can be moderated by job resources [7]. In this section we present the conceptual and empirical foundations for our claim that job resources constitute a multidimensional construct (H1) that moderate the negative effect of work intensification on the PDW (H2).

An early study on job resources with a sample of higher education professionals found that work overload, physical and emotional demands, and work–family conflict did not result in high levels of burnout in those employees who experienced autonomy, social support, and high-quality relationships with their direct supervisor [39]. Likewise, a study conducted with workers of an electrical engineering and electronics company, yielded that exhaustion was mitigated by job resources such as autonomy, control, support and professional development [40]. Interestingly, increase of job demands that comes along with support by leaders and colleagues seem to result in higher levels of work satisfaction [41].

Sonnentag and Fritz [7] state that managers play a critical role in facilitating detachment in order to buffer job stressors that hinder productivity and negatively impact individuals’ health. Nielsen et al., [42] suggest that managers (today’s equivalent of C-level executives) should define and maintain resources that lessen job demands impact. An example of the latter is a safety climate approach [43,44] which consists of practices aimed at preserving the psychological well-being by means of strengthening resources for detachment.

In addition to the establishment of safety climate practices, positive effects of detachment require collaborators’ perception that job resources are available. Consequently, we propose that individuals who perceive high levels of support from their work context, achieve greater levels of PDW. On the contrary, lacking resources, as well as low perceived support is expected to strengthen the negative effects of stressors on PDW [7,15,16,44].

**Figure 1:**
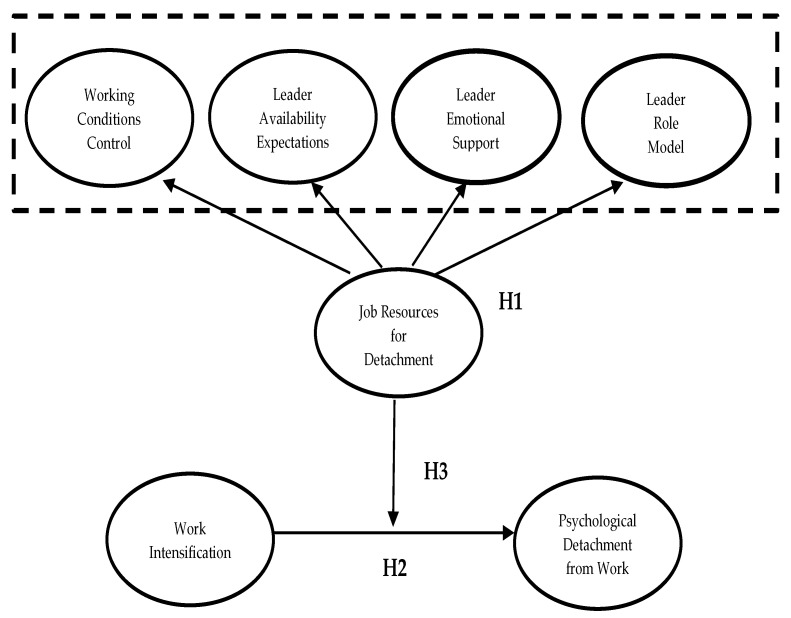
Hypothesized model.

According to the latter we propose:

**Hypothesis** **3:** *Job resources moderate the negative effect of work intensification on PDW, as depicted in* Figure 1.

## 2. Materials and Methods

### 2.1. Participants and Procedure

We developed a scale to assess the perception of the availability of job resources for detachment. Based on the literature (e.g., Mellner et al. [45,46]; Korunka and Gerdenitsch [47]; Bennett et al. [48]) we generated 24 items to measure four constructs that define organizational resources for detachment. After obtaining their informed consent, employees were asked to rate on a scale ranging from 1 to 5 (1 = never, 5 = very frequently), the extent to which they agreed with each one of the statements.

In order to examine the content validity of the items, the instrument was initially administered to a sample of 74 Masters’ in Business Administration students who had a full-time job. The average age was 39.6 years (SD = 8.34); the average work experience was 16.8 years (SD = 8.69), and the average tenure in their current position was 4.9 years (SD = 3.84). Finally, the majority of the participants were women (59%), and 58% of respondents were in a management position.

The construct validity of the final scale was examined with a second sample of workers in a private health organization in Bogotá–Colombia. After obtaining the organization’s approval to conduct the study, we contacted 1425 people via email, and requested them to complete the instrument in question, as well as two additional scales. The response rate was 28% (394 individuals). The participants were mostly women (75.4%), average age was 35.8 years (SD = 8.9), average job tenure was 13.8 years (SD = 8.2), 47.7% had a supervisory position, 59.9% were married, and 57.6% had children. All participants worked full-time, and 67.5% were administrative staff. No exclusion criteria were defined, and the sample to test H2 and H3 was the same as previously described.

### 2.2. Measures

In order to assess work intensification, we used five items of the Intensification of Job Demands Scale [33]. Items evaluate perceptions on changes of work demands (e.g., “… the time between the more intense work phases has decreased”) and involve a rating scale from 1–5 (1 = no at all, 5 = completely). For the present study, we conducted a back-translation process into a Spanish version [49].

PDW was measured with 3 items of the Spanish version of the Recovery Experience Questionnaire [50] (e.g., “I distance myself from work”) on a rating scale from 1–5 (1= I totally disagree, 5= I totally agree).

Job Resources for Detachment was measured with our scale described in Table 1.

### 2.3. Statistical Analyses

In order to analyze the factor structure of the Job Resources for Detachment Scale, we conducted confirmatory factor analyses (CFA), based on maximum likelihood estimation and robust analysis [51]. The quality of the models was tested using chi-square statistics, the goodness-of-fit index (GFI), the no normed fit index (NNFI), and the root mean square error of approximation (RMSEA). GFI and NNFI values close to 0.95 and above, as well as RMSEA values of 0.06 or lower, were assumed to indicate a good fit for the model [52]. Chi-square difference scores (Δχ^2^) were used to compare the models. The previous analyses were conducted in LISREL 8.80.

To specify the reflective-formative structure of Job Resources for PDW as a second order construct (SOC), we used the extended repeated indicators method (PLS-SEM), where all indicators of the lower-order components are assigned to a higher-order component. The extended repeated indicators approach produces smaller biases in the estimation of the higher order construct’s measurement model (i.e., the relations between lower- and higher-order components) [53,54]

In addition, we used the B Mode (PLS-SEM) to estimate formatively specified measurement models. Becker et al., [55] showed that this measurement for repeated indicators applies to the orientation of the higher-order components, instead of the lower-order components. Moreover, their simulation study showed that the B Mode estimation of the higher-order component in a reflective-formative type produced the smallest parameter estimation bias. Finally, the formative model was evaluated based on convergent validity, collinearity, statistical significance, and relevance of the indicator weights [56].

We conducted variance-based structural equation modeling (PLS-SEM). This approach to SEM emphasizes prediction in estimating both the measurement model and the structural model [57]. The measurement model was reflective, meaning that the indicators mirror the constructs. As the moderating variable is a formative construct, we took the two-stage approach [58]. Initially, we calculated the interaction term based on the latent variable scores. Then, we used these scores as single indicators of composites in the model. In order to attain the study objectives, the interaction term, as well as the latent variable scores of IDT and JRPDW were used as independent variables in a multiple linear regression on the latent variable scores of PDW. All previous analyses were performed using SmartPLS 3.3.2. [59]

## 3. Results

The dimensions of the instrument were reduced through communalities extraction. In other to match literature recommendations, items with values below 0.5 were removed [60], as well as those lower than 0.35 in the total item correlation [61]. The final scale consists of 17 items distributed as follows: 4 measuring control over working conditions (α = 0.85); 4 low availability culture (α = 0.91); 4 leader’s emotional support (α = 0.91), and 5 leader’s role model (α = 0.92) (see Table 1).

The CFA showed a good fit for a four-factor model (χ^2^ = 166.96; df = 106: GFI = 0.95; NNFI = 0.99; CFI = 0.10; RMSEA = 0.028; SRMR = 0.04). We compared this four-factor model with alternative two-factor and three-factor models. These models resulted in an acceptable but slightly worse fit than the four-factor model. Table 2 shows the fit indexes comparison. The final scale was composed of 17 items and four factors as proposed in H1. Table 1 displays items wording, descriptive statistics, and factor loadings for Job Resources for Detachment scale.

The validation of the formative model according to Sarstedt et al., [54] will be presented as follows. A convergent validity analysis was conducted by means of testing the redundancy of the Job Resources for Psychological Detachment from Work scale (JRPDW) with an alternative measurement of a single item on culture of care of individuals belonging to the organization. The analysis yielded a path correlation of 0.968 between the higher-order component and the single item latent variable score. The bootstrapping model (5000 subsamples) indicated a lower level of confidence interval (LLCI) 0.960 and a upper level (ULCI) 0.973 range in a 95% CI. This result supports the convergent validity of JRPDW as the path coefficient is superior to the 0.70 threshold.

Second, collinearity was evaluated among the four factors of the model. Results show minimal collinearity with a variance inflation factor (VIF) of all latent variables ranging between 1.40 and 2.00, far below the common cut-off threshold of 5 (see Table 3).

Third, we analyzed the statistical significance and result’s weights. The model’s bootstrapping (5000 subsamples) between the low and the high order constructs yields’ significant values. These relationships are shown as path coefficients but reflect the model’s weights of the JRPDW construct [56]. Although such weights are not high (>0.50), they are significant and the correlations of the variables with the high order construct are significant as well. Table 3 displays the results of the formative model.

As shown in Table 4, composite reliability coefficients reached values above 0.70. Convergent validity was assessed for constructs, as well as for observed values. The average variance extracted (AVE) was superior to 0.60, the VIF lesser than 0.5, and outer loadings of observed indicators greater than 0.6, thus providing evidence for appropriate consistency and validity levels [62]. Since the JRWD is a formative construct, we were not able to obtain validity or reliability indicators.

We adopted two discriminant validity criteria. First, we evaluated whether the square root of the AVE was greater than the correlation of both constructs [62]. Second, we conducted an heterotrait-monotrait (HTMT) ratio of correlations assessment, were values inferior to 0.85, with confidence intervals different than zero are considered as appropriate. Results for both criteria suggest the constructs of the model have good levels of discriminant validity. Given the unavailability of the AVE and the HTMT ratio of correlations, we were unable to assess the discriminant validity of the formative construct (see Table 5).

Table 6 displays the values of the estimation of the structural model (bootstrap 5000 samples) which yields a direct negative effect (−0.155) of WI on PDW, and a positive effect (0.460) of JRPDW on PDW. Both constructs explain 30,2% of the variance of PDW. The effect size for WI on PDW was weak ƒ^2^ > 0.026, and for JRPDW on PDW was high ƒ^2^ > 0.230 [63]. Finally, predictive performance of PDW was moderate Q^2^ = 0.246, [56].

Once we had identified the latent variable scores, the next step involved estimating the model while including the effect of the interaction between the independent variable and the moderating variable. Given that in PLS-SEM, all variables are standardized, the interaction is interpreted as the change of the conditioned effect of WI on PDW when JRPDW varies by one standard deviation.

The estimation of the structural model yields an unconditional negative effect (−0.103) of WI on PDW (*t* = 2.018; *p* ≤ 0.022; [−0.191, −0.022], and an unconditional positive effect (0.451) of JRPDW on PDW (*t* = 9.399; *p* ≤ 0.000; [0.369, 0.526]. The interaction indicates a conditional effect (−0.82) of WI on PDW when JRPDW changes (*t* = 1.734; *p* ≤ 0.041; [−0.159, −0.004].

**Figure 2:**
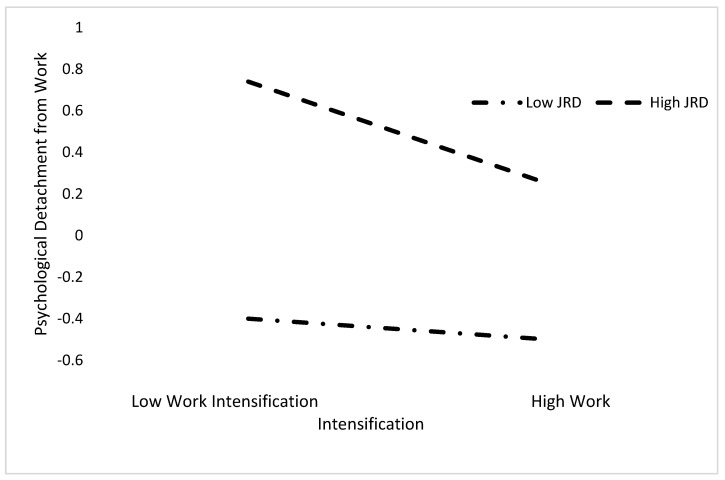
Relationship between work intensification and psychological detachment from work as a function of Job Demands Resources (JD-R) high/low values correspond to 1 SD above/below mean.

Figure 2 represents the interaction effect between WI and JRPDW and shows that higher levels of perceived resources are related to higher levels of psychological detachment. These results provide empirical support for H2 and H3 regarding the moderating effect of JRPDW between demands intensification and psychological detachment from work.

## 4. Discussion

The purpose of the present study was twofold. First, we intended to develop an instrument to evaluate job resources. Second, we aimed to identify the relationship between such resources and psychological detachment.

According to the data, our instrument comprises the following four-factor structure: Control over working conditions, Low availability culture, Leader’s emotional support, and Leader’s role model. Moreover, the abovementioned factors form a second order model. As previously stated, most of the literature has focused on individual, instead of organizational resources. In this regard, this study entails a broader approach to organizational resources and their ability to facilitate detachment. In addition, our results suggest the implication of multiple factors in job resources. Compared with previous research, this study points to more complex interactions of variables, which will be discussed as follows.

Consistent with the hypothesis, job resources moderate the relationship between work intensification and psychological detachment. As far as we know, there is no previous evidence for such relationship. Results indicate that perceiving higher levels of support from the work context facilitates higher levels of psychological detachment. Conversely, individuals who perceive lower levels of support have a decreased chance of detaching from work. In our opinion, one of the most noteworthy results is that, even in the face of low levels of work intensification, the levels of psychological detachment are low when job resources are low. This finding serves to underline the weight of job resources on individuals’ potential to distance themselves from work. From an applied perspective, the latter means that individuals (and organizations) may benefit from making available job resources even in the absence of heightened work demands. Overall, findings are in line with theories that contend that coping strategies mitigate stress [7,64,65,66]. Similarly, the study provides empirical support for the notion that intensification is inversely related to detachment [10].

Furthermore, our findings are consistent with the idea that social acceleration intensifies work, which in turn affects psychological well-being [35]. Finally, the results are congruent with the concept that the effects of work intensification are fundamentally negative as they interfere with the process of recovery [1]. This points to the need for promoting a balance between short-term organizational benefits of work intensification, and the deleterious consequences associated with workers’ health and well-being.

Several levels of explanation may account for the moderating effect of resources between intensification and psychological detachment. First, instrumental resources may endow workers with autonomy to define working times, technology use, and extension of their working day [36,38]. Consequently, organizations may contribute to detachment by promoting control over objective planning, task tracking, and definition of limits between personal and work roles [67]. In our instrument, these resources are clustered in control over working conditions.

Second, social resources involve emotional aid received from leaders in order to meet job demands and facilitate detachment. The latter are related to expectations of leader’s availability and role modeling. Our findings provide evidence on the importance of perceived segmentation and integration preferences in the organization [68,69]. In the same vein, leadership roles defining management models in favor of segmentation may help create a suitable sense of psychological detachment and achieve adequate recovery levels. Hence, it depends on organization’s managers to promote a culture underpinned by values of respect for the separation of roles, and the establishment of work–life balance [7,16,28].

Finally, results indicate that job resources interact in their protective effect from work intensification. Along these lines, our study suggests complex dynamics between individuals and organizations. This is consistent with Derks et al., [70] findings that burnout, associated with a highly demanding work, was buffered in individuals with high-quality relationships with their supervisors, social support and autonomy, among other variables.

In our view, there are three limitations to highlight in the study. First, the cross-sectional design interferes with the possibility to identify causal relationships amidst the variables. However, such relationships were beyond the scope of the study as we initially intended to explore the association of job resources with psychological detachment. Second, self-reports could have led to bias of common variance. Nonetheless, the use of advanced methodological procedures based on confirmatory factor analyses and structural equation modeling confers validity to the results [71]. Finally, we would like to point that the results may not be generalized to other populations. However, we presume that boundaryless conditions at work are pervasive across different contexts and professions.

## 5. Conclusions

According to the data, job resources are multidimensional in nature. In addition, they moderate the relationship between intensification and psychological detachment, which is congruent with the stressor-detachment model (SDM). The main organizational implication is that culture in work contexts plays an important role in either facilitating or interfering with psychological detachment. Moreover, job resources may interact with each other and potentiate their protective effect. If this is the case, organizations may better promote detachment by making available multiple instead of single resources.

From an applied standpoint, the scale constitutes a tool to evaluate job resources in different contexts, by both scholars and practitioners. To the best of our knowledge, this is the first instrument devised to assess this construct. We hope this study stimulates further research involving complex interactions between individual and organizational resources from multidimensional perspectives.

## Figures and Tables

**Table 1:** Means, SD, corrected item-total correlation and factor loadings.

Item	M	SD	ITC	1	2	3	4
In this organization							
My tasks and responsibilities require that I work even during off-work time	3.06	1.17	0.49	0.74			
It is natural for me to finish work at home	2.46	1.18	0.55	0.91			
It is accustomed to use work-related Applications in personal mobile devices	2.86	1.46	0.65	0.93			
Work matters are taken care of any time, any place	2.50	1.24	0.41	0.78			
In this organization leaders:							
Value more those who respond to their messages even in non-workdays	3.26	1.18	0.70		0.60		
Hope that people extend their regular working time	2.96	1.21	0.81		0.87		
Contact people beyond their regular working time	3.33	1.14	0.81		0.86		
Assign work beyond their regular working time	2.65	1.11	0.80		0.83		
Are willing to ease the work-family balance	3.25	1.20	0.60			0.98	
Are interested to know my family and personal needs	3.04	1.19	0.77			0.99	
Are open to discuss possible work-family conflicts	3.23	1.15	0.78			0.97	
Help me solve such conflicts	3.04	1.09	0.66			0.94	
My leader:							
Is a role-model from whom to learn how to balance work and life	3.14	1.15	0.85				0.90
Is a role-model; personally, and professionally	3.21	1.09	0.85				0.84
Does not deal with work matters when enjoying time off work	3.13	1.05	0.68				0.74
Plans work according to the team’s personal and family needs	3.08	1.10	0.77				0.90
Motivates workers to leave work matters behind, right after working hours	2.98	1.12	0.79				0.94

Note. M = mean; SD = standard deviations; ITC = item-test total correlations; 1 = control over the extension of working hours; 2 = expectations of the leader’s availability; 3 = emotional support from the leader; 4 = leader’s role modeling.

**Table 2:** Goodness of fit statistics.

Models	χ^2^	df	GFI	NNFI	CFI	RMSEA	SRMR
One-factor model	1378.52	119	0.61	0.75	0.78	0.18	0.16
Best fitting two-factor model ^a^	1021.96	118	0.71	0.85	0.87	0.14	0.14
Best fitting three-factor model ^b^	311.50	109	0.91	0.97	0.98	0.061	0.05
Four-factor model	166.96	106	0.95	0.99	0.10	0.028	0.04

Note. GFI = goodness-of-fit index; NNFI = no normed fit index; CFI = comparative fix index; RMSEA = root mean square error of approximation; SRMR = standardized root mean square residual. ^a^ Control over working conditions items load in the first factor. Low availability culture items, leader’s emotional support and leader’s role model items load in the second factor. ^b^ Control over working conditions items load in the first factor. Low availability culture items load in the second factor. Leader’s emotional support, and leader’s role model load in the third factor.

**Table 3:** Second order construct estimates.

Low Order Construct	VIF	Weights	*p* Value	Correlation with JRPDW
Control Over Working Conditions–COW	1.429	0.327	0.000	0.605 **
Low availability culture–LAC	1.550	0.318	0.000	0.716 **
Leader’s emotional support–LES	1.917	0.356	0.000	0.739 **
Leader’s role model–LRM	1.995	0.389	0.000	0.798 **

Note. ** *p* < 0.05; VIF = variance inflation factor; JDRPW = job resources psychological detachment from work.

**Table 4:** Measurement model statistics.

Variable	Outer Loadings	VIF	Rho-A	CR	AVE	Q^2^ Predict
Intensification Job Demands			0.829	0.852	0.539	
IDT1	0.819 ***	1633				
IDT2	0.801 ***	1965				
IDT3	0.711 ***	1348				
IDT4	0.749 ***	1481				
IDT5	0.673 ***	1424				
Job Resources for PDW			n.a	n.a	n.a	
Psychological Detachment from Work			0.892	0.932	0.821	0.246
PDW1	0.894 ***	2435				0.191
PDW2	0.916 ***	2793				0.216
PDW3	0.909 ***	2707				0.198

Note. *** *p* < 0.000; VIF = variance inflation factor; rho_A = Spearman’s Rho; CR = composite reliability; AVE = average variance extracted; Q^2^ *predict* = predictive performance.

**Table 5:** Discriminant validity of the measurement model.

Construct	IDS	JRPDW	PDW
Work Intensification–WI	0.753	n.a	0.369 [0.262, 0.469]
Job Resources for Detachment–JRPDW	−0.451	n.a	n.a
Psychological Detachment from Work–PDW	−0.329	0.507	0.906

Note. On diagonal, square root of AVE; correlation between constructs presented below the diagonal; HTMT present above diagonal; numbers in brackets represent the 95% bias-correct and accelerated confidence intervals derived from bootstrapping with 5000 samples.

**Table 6:** Structural model estimates.

Relationships	Path Coefficient	*t*-Statistic	*p*-Value	95% CI BCa	ƒ^2^
WI → PDW	−0.155	2.771	0.006	[−0.257, −0.039]	0.026
JRPDW → PDW	0.460	8.526	0.000	[0.340, 0.522]	0.230

Note. 95% CI BCa = 95% bias-corrected and accelerated confidence intervals; f^2^ = effect size.

## Data Availability

The data presented in this study are available on request from the corresponding author.
